# Supplementary material for: Simultaneous epigenomic profiling and regulatory activity measurement using e2MPRA
Source: Nat Commun. 2026 Jan 14;17:1724. doi: 10.1038/s41467-026-68422-3 (PMC12913623; doi:10.1038/s41467-026-68422-3)
Supplement: Supplementary file 1 — Supplementary Information [file 41467_2026_68422_MOESM1_ESM.pdf]

Supplementary Figures

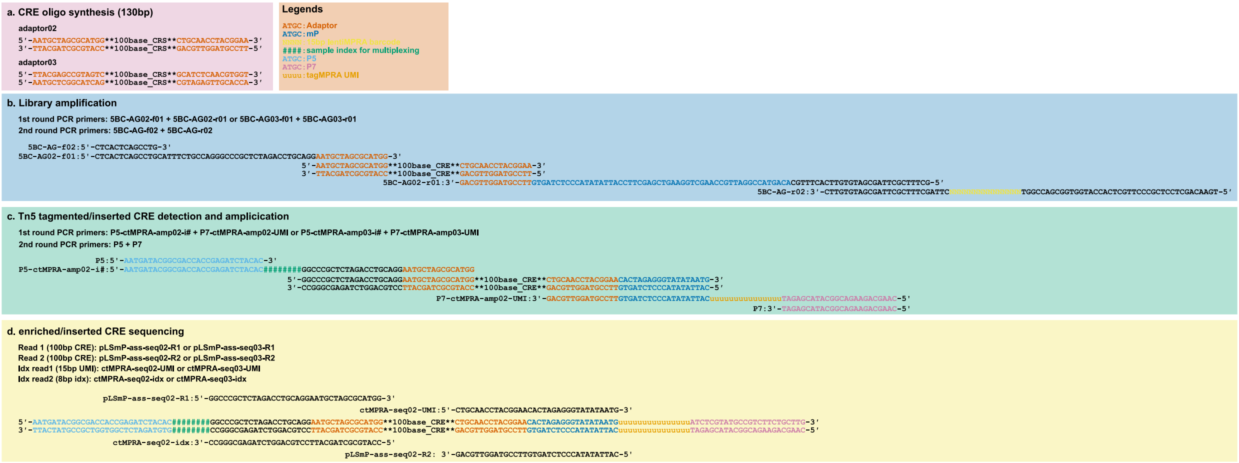

Supplementary Fig 1. Sequence scheme of e2MPRA. a, Structure of the synthesized CRE oligonucleotides. For each of the three libraries, a distinct pair of 15-bp adapter sequences was added to both ends of the CRE. **b**, Primer sequences and their corresponding binding sites used in the first and second rounds of PCR for library amplification. **c**, Primer sequences and their binding sites used to amplify CRE fragments following ATAC or CUT&Tag. **d**, Primer sequences and their binding sites used for sequencing to quantify the amplified CRE fragments.

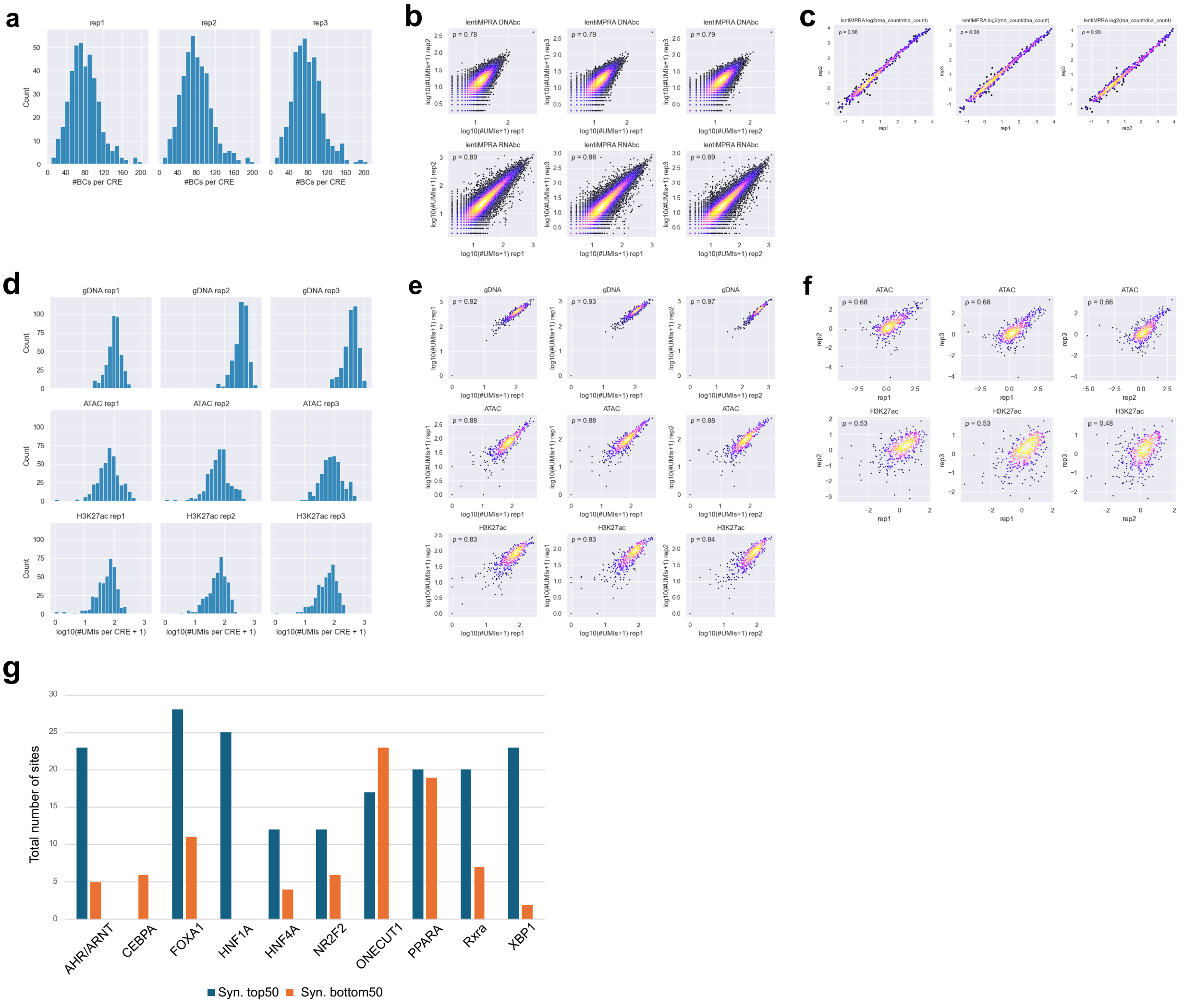

Supplementary Fig 2. Comparison of barcode counts, enriched counts, and inserted CRE counts across replicates for the pilot library. a, Distribution of barcode coverage per CRE in each lentiMPRA replicate. **b**, Scatter plot showing the correlation of the number of UMIs for DNA and RNA barcodes in lentiMPRA between replicates. This reflects the raw count-level reproducibility of each measurement. Spearman's ρ is shown in the upper left. **c**, Scatter plot showing the correlation of $\log_2$(RNA barcode count / DNA barcode count) between replicates. Spearman's correlation coefficient (ρ) is shown in the upper left. **d**, Distribution of inserted CRE counts (from gDNA) and enriched CRE counts (from ATAC and H3K27ac CUT&Tag) per CRE in each replicate. **e**, Scatter plot showing the correlation of inserted and enriched CRE counts between replicates. This reflects the raw count-level reproducibility of each measurement. Spearman's ρ is shown in the upper left. **f**, Scatter plot showing the correlation of $\log_2$(enriched CRE count / inserted CRE count) between replicates. This represents the reproducibility of the normalized epigenetic activity scores. Spearman's ρ is shown in the upper left. **g**, A bar plot showing the difference in the total number of transcription factor binding sites between the top 50 and bottom 50 synthetic sequences.

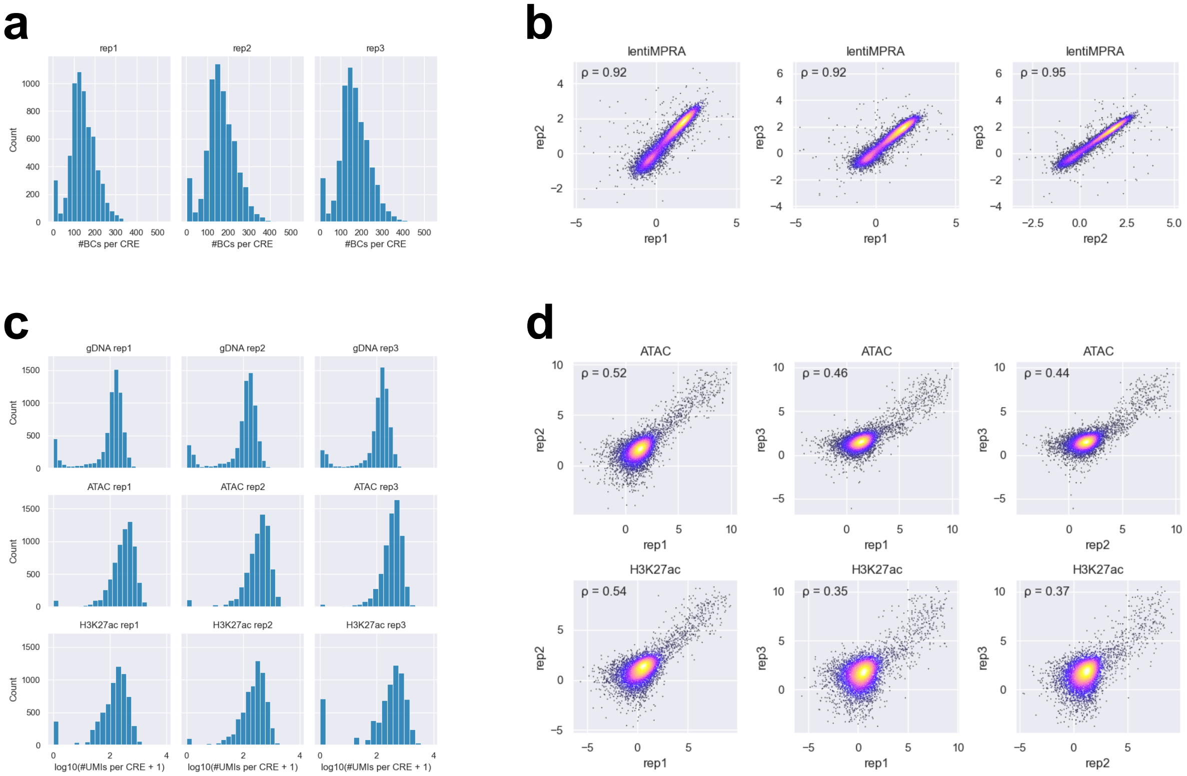

Supplementary Fig 3. Comparisons of barcode counts, enriched counts, and inserted CRE counts across replicates for the HepG2 library. a, Distribution of barcode coverage per CRE in each lentiMPRA replicate. **b**, Scatter plot showing the correlation of $\log_2$(RNA barcode count / DNA barcode count) between replicates. Spearman's correlation coefficient (ρ) is shown in the upper left. **c**, Distribution of inserted CRE counts (from gDNA) and enriched CRE counts (from ATAC and H3K27ac CUT&Tag) per CRE in each replicate. **d**, Scatter plot showing the correlation of $\log_2$(enriched CRE count / inserted CRE count) between replicates. This represents the reproducibility of the normalized epigenetic activity scores. Spearman's ρ is shown in the upper left.

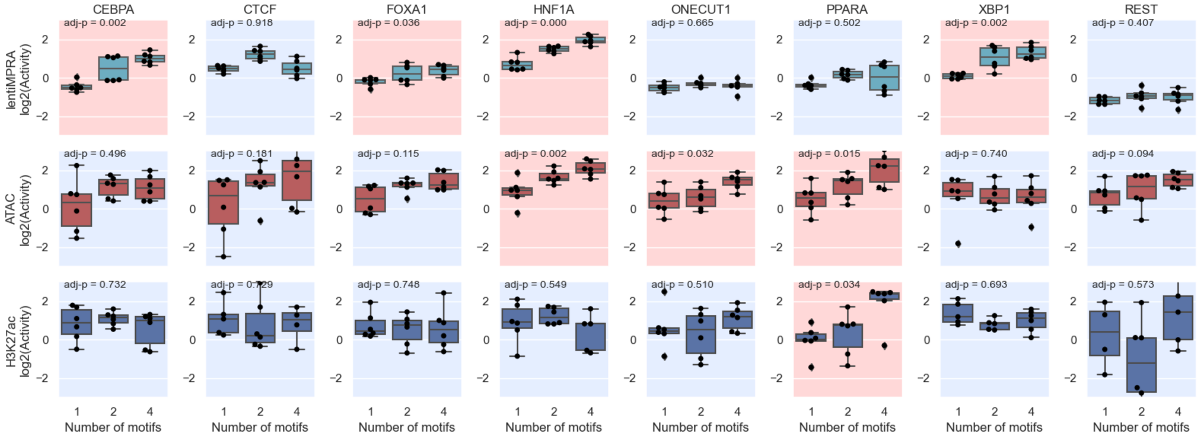

Supplementary Fig 4. Epigenetic activities measured for Class 1 sequences across all TF binding motifs. Significant Spearman's correlations (FDR < 0.05) between TFBS copy number (x-axis) and log2(Activity) (y-axis) are indicated with a red background and non-significant correlations are indicated with a blue background. Two-sided Spearman rank correlation tests were performed, and p-values were adjusted using the Benjamini–Hochberg correction. n = 2 templates × 3 replicates per box plot. In the box plots, the median is indicated by the black line; the lower and upper bounds of the box represent the first (25th percentile) and third (75th percentile) quartiles, respectively; and the lower and upper whiskers indicate the minimum and maximum values within 1.5 × the interquartile range (IQR).

a

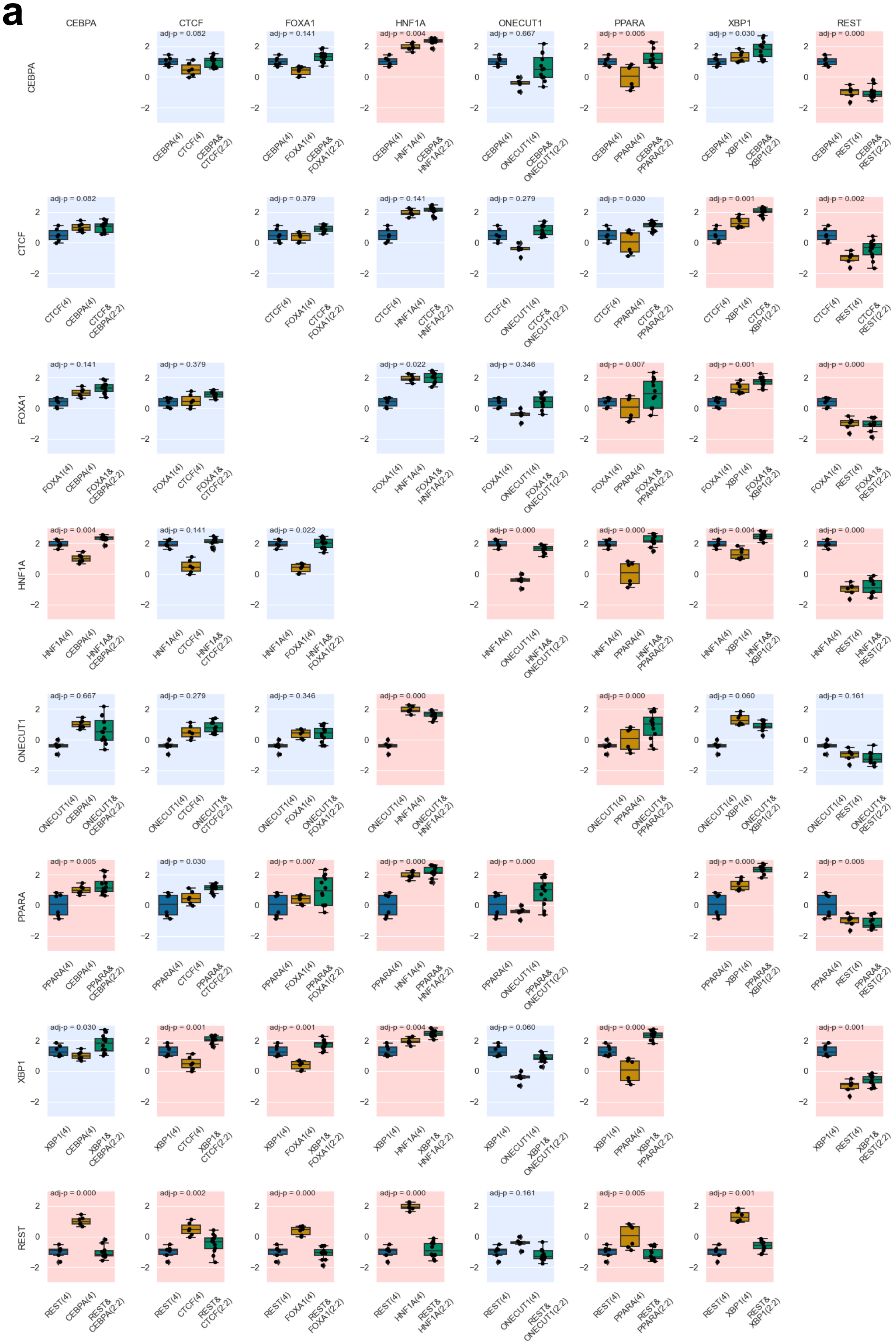

CEBPA
CTCF
FOXA1
HNF1A
ONECUT1
PPARA
XBP1
REST
CEBPA: adj-p = 0.082; adj-p = 0.141; adj-p = 0.004; adj-p = 0.667; adj-p = 0.005; adj-p = 0.030; adj-p = 0.000
CTCF: adj-p = 0.082; adj-p = 0.379; adj-p = 0.141; adj-p = 0.279; adj-p = 0.030; adj-p = 0.001; adj-p = 0.002
FOXA1: adj-p = 0.141; adj-p = 0.379; adj-p = 0.022; adj-p = 0.346; adj-p = 0.007; adj-p = 0.001; adj-p = 0.000
HNF1A: adj-p = 0.004; adj-p = 0.141; adj-p = 0.022; adj-p = 0.000; adj-p = 0.000; adj-p = 0.004; adj-p = 0.000
ONECUT1: adj-p = 0.667; adj-p = 0.279; adj-p = 0.346; adj-p = 0.000; adj-p = 0.000; adj-p = 0.060; adj-p = 0.161
PPARA: adj-p = 0.005; adj-p = 0.030; adj-p = 0.007; adj-p = 0.000; adj-p = 0.000; adj-p = 0.000; adj-p = 0.005
XBP1: adj-p = 0.030; adj-p = 0.001; adj-p = 0.001; adj-p = 0.004; adj-p = 0.060; adj-p = 0.000; adj-p = 0.001
REST: adj-p = 0.000; adj-p = 0.002; adj-p = 0.000; adj-p = 0.000; adj-p = 0.161; adj-p = 0.005; adj-p = 0.001

b

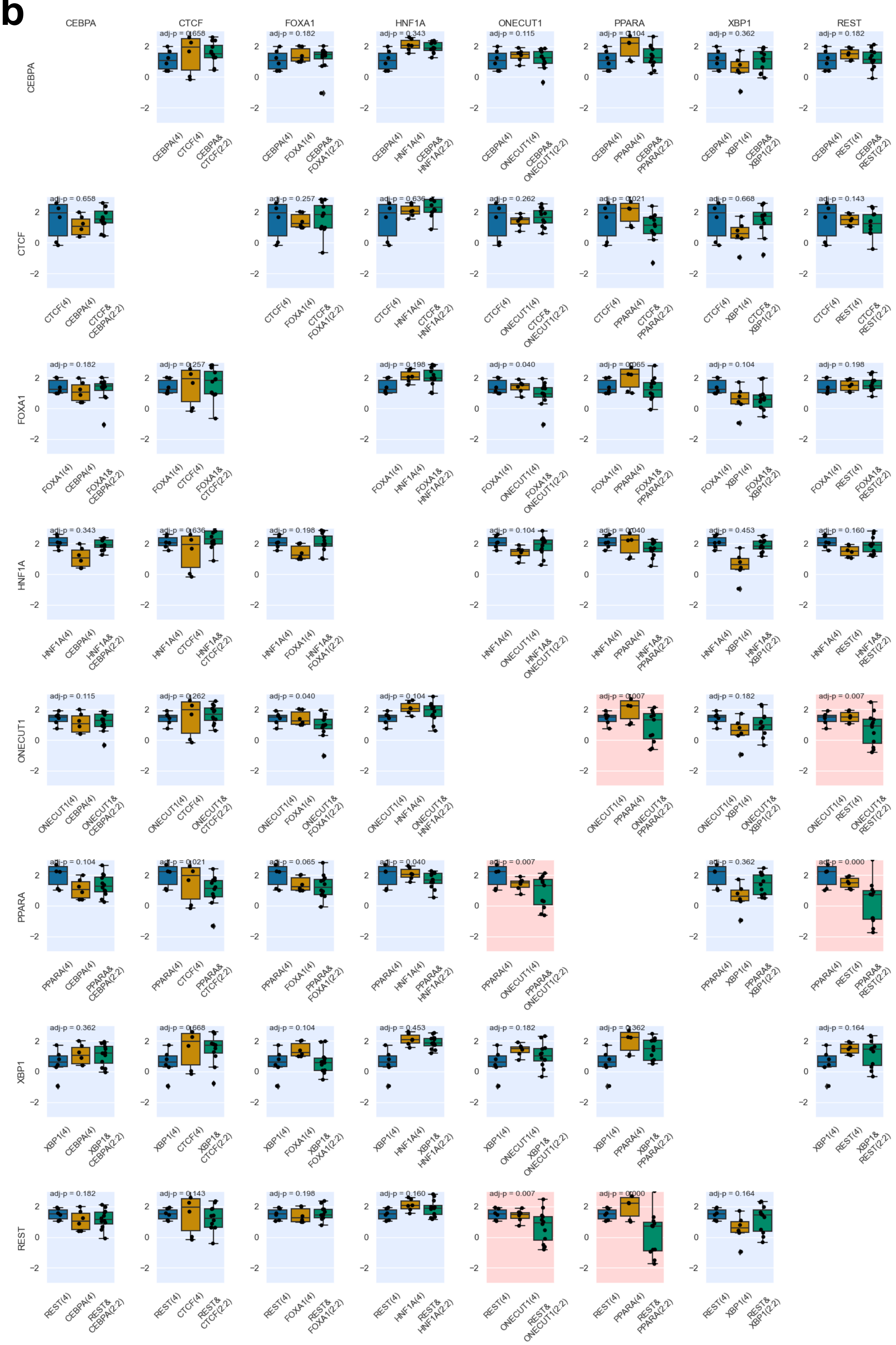

CEBPA
CTCF
FOXA1
HNF1A
ONECUT1
PPARA
XBP1
REST
CEBPA: adj-p = 0.658; adj-p = 0.182; adj-p = 0.343; adj-p = 0.115; adj-p = 0.104; adj-p = 0.362; adj-p = 0.182
CTCF: adj-p = 0.658; adj-p = 0.257; adj-p = 0.636; adj-p = 0.262; adj-p = 0.021; adj-p = 0.668; adj-p = 0.143
FOXA1: adj-p = 0.182; adj-p = 0.257; adj-p = 0.198; adj-p = 0.040; adj-p = 0.065; adj-p = 0.104; adj-p = 0.198
HNF1A: adj-p = 0.343; adj-p = 0.636; adj-p = 0.198; adj-p = 0.104; adj-p = 0.040; adj-p = 0.453; adj-p = 0.160
ONECUT1: adj-p = 0.115; adj-p = 0.262; adj-p = 0.040; adj-p = 0.104; adj-p = 0.007; adj-p = 0.182; adj-p = 0.007
PPARA: adj-p = 0.104; adj-p = 0.021; adj-p = 0.065; adj-p = 0.040; adj-p = 0.007; adj-p = 0.362; adj-p = 0.000
XBP1: adj-p = 0.362; adj-p = 0.668; adj-p = 0.104; adj-p = 0.453; adj-p = 0.182; adj-p = 0.362; adj-p = 0.164
REST: adj-p = 0.182; adj-p = 0.143; adj-p = 0.198; adj-p = 0.160; adj-p = 0.007; adj-p = 0.000; adj-p = 0.164

c

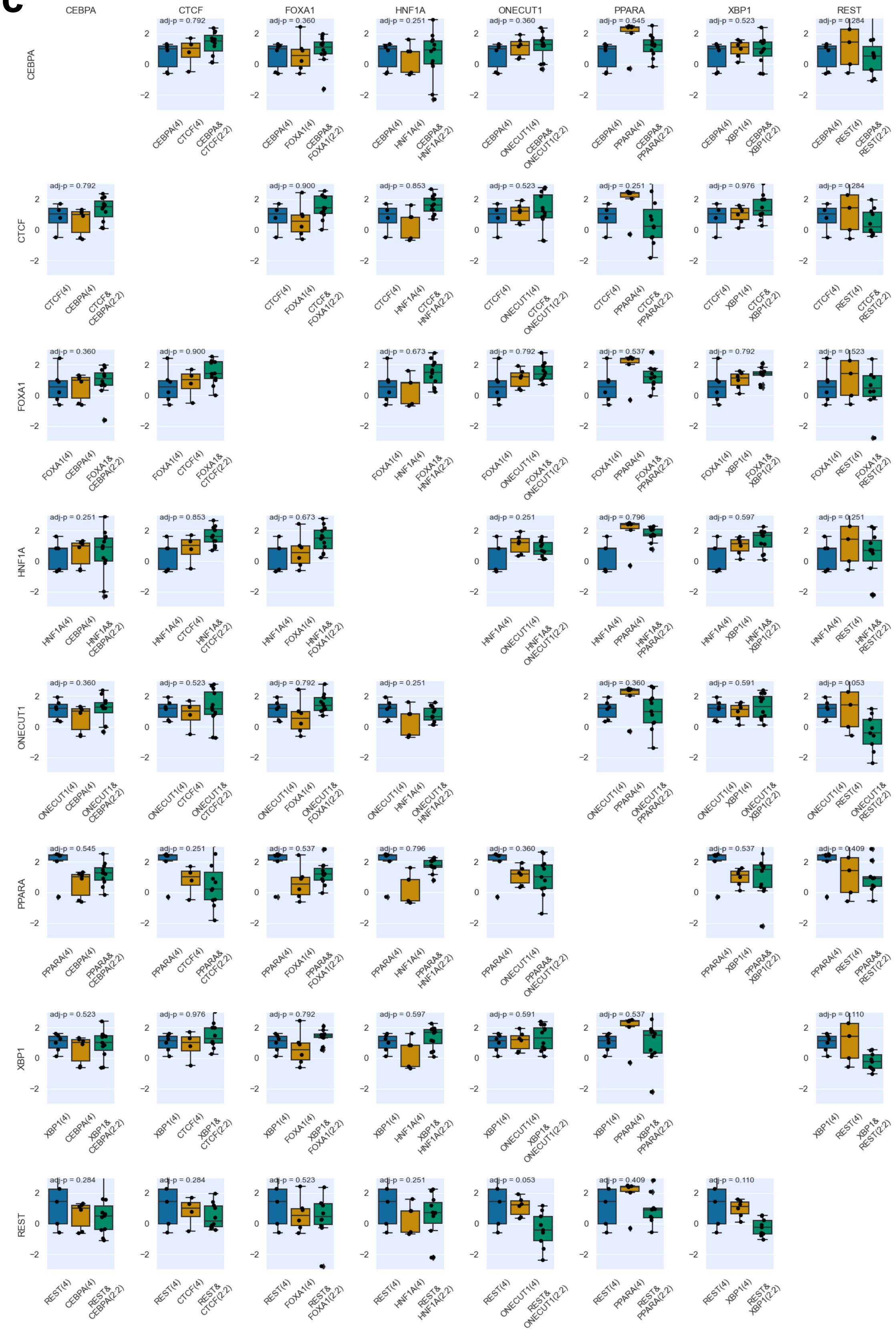

Supplementary Fig 5. Epigenetic activities measured for Class 2 sequences across all TF binding motif pairs. **a**, lentiMPRA; **b**, ATAC-seq; **c**, H3K27ac CUT&Tag. Transcriptional or epigenetic activities of heterotypic TFBS arrangements (Class 2; two different motifs combined in a 2:2 ratio) were compared to homotypic arrangements (Class 1; four identical motifs) to assess synergistic effects. Statistically significant synergistic interactions, as determined by linear regression and two-sided t-tests with Benjamini–Hochberg correction (FDR < 0.01), are indicated by a red background and non-significant combinations are shown with a blue background. n = 2 templates × 3 replicates per box plot. In the box plots, the median is indicated by the black line; the lower and upper bounds of the box represent the first (25th percentile) and third (75th percentile) quartiles, respectively; and the lower and upper whiskers indicate the minimum and maximum values within 1.5 × the interquartile range (IQR).

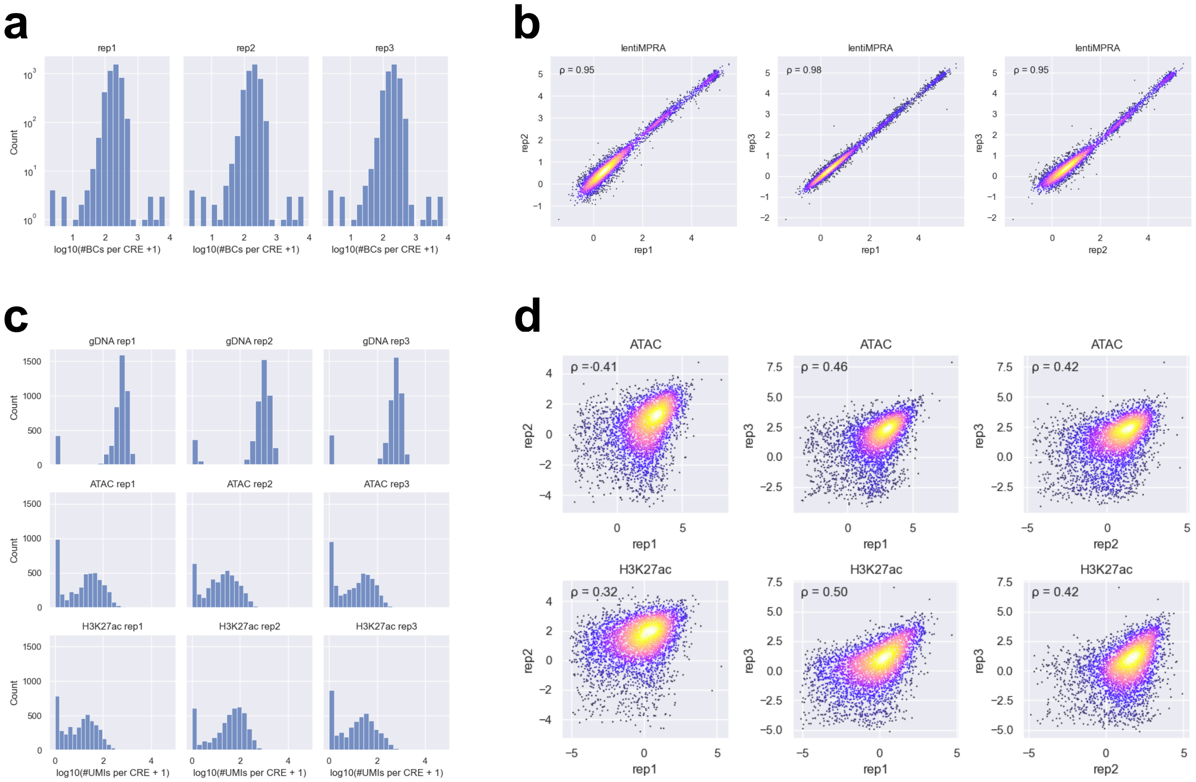

Supplementary Fig. 6. Comparisons of barcode counts, enriched counts, and inserted CRE counts across replicates for the WTC11 library. a, Distribution of barcode coverage per CRE in each lentiMPRA replicate. **b**, Scatter plot showing the correlation of $\log_2$(RNA barcode count / DNA barcode count) between replicates. Spearman's correlation coefficient (ρ) is shown in the upper left. **c**, Distribution of inserted CRE counts (from gDNA) and enriched CRE counts (from ATAC and H3K27ac CUT&Tag) per CRE in each replicate. **d**, Scatter plot showing the correlation of $\log_2$(enriched CRE count / inserted CRE count) between replicates. This represents the reproducibility of the normalized epigenetic activity scores. Spearman's ρ is shown in the upper left.

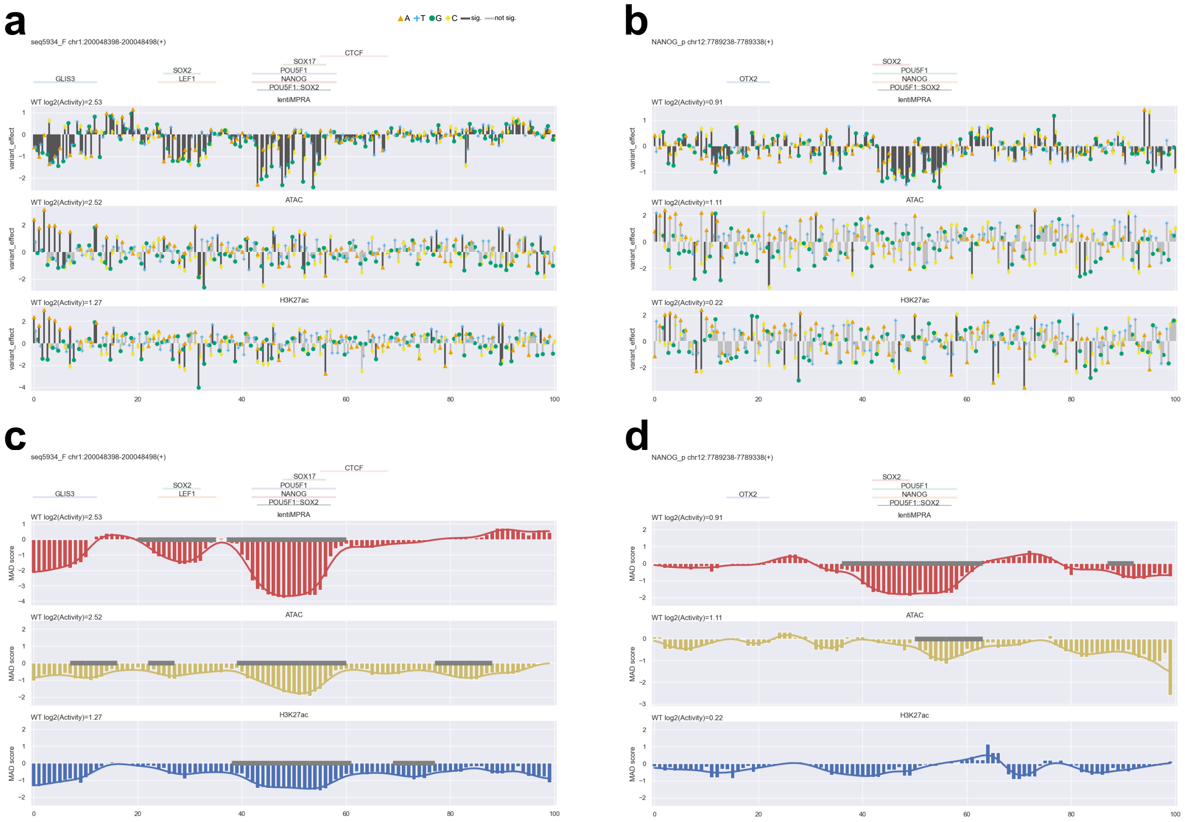

Supplementary Fig. 7. CRE perturbation analyses for the remaining two CREs not shown in Fig. 5. **a-b**, Analysis of single-nucleotide substitution effects on epigenetic activities (lentiMPRA, ATAC, and H3K27ac CUT&Tag) for seq5934_F (**a**) and NANOG_p (**b**). Annotated TF motifs[22] are shown above each plot. Bar colors represent statistical significance (black: significant at $P < 0.01$; gray: not significant). P values were obtained from multiple linear regression. **c-d**, Analysis of 6-bp window perturbation effects for seq5934_F (**c**) and NANOG_p (**d**). Positional effects of mutations were quantified as median absolute deviation (MAD) scores at each nucleotide position and smoothed using a Gaussian filter (line plot). Regions significantly affected by mutations, as identified by edge detection, are indicated by gray lines along the baseline.
