# Supplementary material for: Simultaneous epigenomic profiling and regulatory activity measurement using e2MPRA
Source: Nat Commun. 2026 Jan 14;17:1724. doi: 10.1038/s41467-026-68422-3 (PMC12913623; doi:10.1038/s41467-026-68422-3)
Supplement: Supplementary file 2 — Description of Additional Supplementary Information [file 41467_2026_68422_MOESM2_ESM.pdf]

Description of Additional Supplementary Files

Supplementary Data 1. Pilot library design

Supplementary Data 2. HepG2 synthetic enhancer library design

Supplementary Data 3. WTC11 enhancer perturbation library design

Supplementary Data 4. lentiMPRA DNA and RNA counts for the pilot library

Supplementary Data 5. ATAC and CUT&Tag counts for the pilot library

Supplementary Data 6. lentiMPRA DNA and RNA counts for the HepG2 library

Supplementary Data 7. ATAC and CUT&Tag counts for the HepG2 library

Supplementary Data 8. lentiMPRA DNA and RNA counts for the WTC11 library

Supplementary Data 9. ATAC and CUT&Tag counts for the WTC11 library

Supplementary Data 10. Primers used in e2MPRA

Supplementary Data 11. Transcription factor binding motifs used in the HepG2 library
