# Supplementary material for: Simultaneous epigenomic profiling and regulatory activity measurement using e2MPRA
Source: Nat Commun. 2026 Jan 14;17:1724. doi: 10.1038/s41467-026-68422-3 (PMC12913623; doi:10.1038/s41467-026-68422-3)
Supplement: Supplementary file 4 — Reporting Summary [file 41467_2026_68422_MOESM4_ESM.pdf]

nature portfolio

Corresponding author(s): Nadav Ahituv, Fumitaka Inoue

Last updated by author(s): Oct 29, 2025

Reporting Summary

Nature Portfolio wishes to improve the reproducibility of the work that we publish. This form provides structure for consistency and transparency in reporting. For further information on Nature Portfolio policies, see our Editorial Policies and the Editorial Policy Checklist.

Statistics

For all statistical analyses, confirm that the following items are present in the figure legend, table legend, main text, or Methods section.

n/a	Confirmed	
☐	☒	The exact sample size (*n*) for each experimental group/condition, given as a discrete number and unit of measurement
☐	☒	A statement on whether measurements were taken from distinct samples or whether the same sample was measured repeatedly
☐	☒	The statistical test(s) used AND whether they are one- or two-sided<br>*Only common tests should be described solely by name; describe more complex techniques in the Methods section.*
☐	☒	A description of all covariates tested
☐	☒	A description of any assumptions or corrections, such as tests of normality and adjustment for multiple comparisons
☐	☒	A full description of the statistical parameters including central tendency (e.g. means) or other basic estimates (e.g. regression coefficient) AND variation (e.g. standard deviation) or associated estimates of uncertainty (e.g. confidence intervals)
☐	☒	For null hypothesis testing, the test statistic (e.g. *F*, *t*, *r*) with confidence intervals, effect sizes, degrees of freedom and *P* value noted<br>*Give P values as exact values whenever suitable.*
☒	☐	For Bayesian analysis, information on the choice of priors and Markov chain Monte Carlo settings
☒	☐	For hierarchical and complex designs, identification of the appropriate level for tests and full reporting of outcomes
☐	☒	Estimates of effect sizes (e.g. Cohen's *d*, Pearson's *r*), indicating how they were calculated

Our web collection on statistics for biologists contains articles on many of the points above.

Software and code

Policy information about availability of computer code

Data collection	ChIP-Atlas (last accessed: 2023-05-11)
Data analysis	bcl2fastq v2.20, FIMO ver. 5.5.1, MPRAflow v2.3.5, BWA 0.7.18-r1243-dirty, samtools v1.11, UMI-tools v1.1.4, bigWigAverageOverBed v2, https://github.com/ziczhang/e2MPRA_analysis

For manuscripts utilizing custom algorithms or software that are central to the research but not yet described in published literature, software must be made available to editors and reviewers. We strongly encourage code deposition in a community repository (e.g. GitHub). See the Nature Portfolio guidelines for submitting code & software for further information.

Data

Policy information about availability of data

All manuscripts must include a data availability statement. This statement should provide the following information, where applicable:

- Accession codes, unique identifiers, or web links for publicly available datasets
- A description of any restrictions on data availability
- For clinical datasets or third party data, please ensure that the statement adheres to our policy

The e2MPRA sequencing data generated in this study, including association barcode sequencing data and barcode sequencing data for lentiMPRA, as well as ATAC-seq and CUT&Tag-seq data, have been deposited at Zenodo (https://zenodo.org/records/15428846 and https://zenodo.org/records/15469962). Source data are provided with this paper as Supplementary Tables.

Research involving human participants, their data, or biological material

Policy information about studies with human participants or human data. See also policy information about sex, gender (identity/presentation), and sexual orientation and race, ethnicity and racism.

Reporting on sex and gender	N/A
Reporting on race, ethnicity, or other socially relevant groupings	N/A
Population characteristics	N/A
Recruitment	N/A
Ethics oversight	N/A

Note that full information on the approval of the study protocol must also be provided in the manuscript.

Field-specific reporting

Please select the one below that is the best fit for your research. If you are not sure, read the appropriate sections before making your selection.

[x] Life sciences [ ] Behavioural & social sciences [ ] Ecological, evolutionary & environmental sciences

For a reference copy of the document with all sections, see nature.com/documents/nr-reporting-summary-flat.pdf

Life sciences study design

All studies must disclose on these points even when the disclosure is negative.

Sample size	All sample sizes are noted in the manuscript. We generated three sequence libraries for the e2MPRA assay. In the first pilot library, we selected a total of 400 sequences from six categories, with each category containing either 50 or 100 sequences. In the second HepG2 library, we selected nine liver-specific transcription factors, generated all possible combinations and permutations, and assessed their activity using two neutral templates. In the third WTC11 library, we selected nine enhancers that are potentially functional in this cell line, generated all possible single-nucleotide substitution variants, and introduced perturbations in sliding windows of six base pairs.
Data exclusions	Data exclusions are clearly indicated in the manuscript. In the HepG2 library, among the nine transcription factors, sequences containing the NR2F2 motif were not detected in the DNA reads; therefore, all combinations and permutations involving NR2F2 were excluded from the analysis. In the WTC11 library, among the nine enhancers, the sequence seq6846_R was not detected in the DNA reads; consequently, all single-nucleotide substitution variants and perturbation variants of this enhancer were excluded.
Replication	All assays (lentiMPRA, ATAC-seq, and CUT&Tag-seq) were performed in three replicates. The pilot library includes three technical replicates for the HepG2 cell line. The HepG2 and WTC11 libraries each include three biological replicates
Randomization	N/A
Blinding	Blinding was not performed because the identity of each library was required for downstream analysis.

Reporting for specific materials, systems and methods

We require information from authors about some types of materials, experimental systems and methods used in many studies. Here, indicate whether each material, system or method listed is relevant to your study. If you are not sure if a list item applies to your research, read the appropriate section before selecting a response.

Materials & experimental systems

n/a	Involved in the study	
[ ]	[x]	Antibodies
[ ]	[x]	Eukaryotic cell lines
[x]	[ ]	Palaeontology and archaeology
[x]	[ ]	Animals and other organisms
[x]	[ ]	Clinical data
[x]	[ ]	Dual use research of concern
[x]	[ ]	Plants

Methods

n/a	Involved in the study	
[x]	[ ]	ChIP-seq
[x]	[ ]	Flow cytometry
[x]	[ ]	MRI-based neuroimaging

Antibodies

Antibodies used	Anti-H3K27ac (Abcam: ab4729), goat anti-rabbit IgG (Abcam: ab6702)
Validation	Anti-H3K27ac (ab4729, Abcam) is marketed as ChIP-grade and has been extensively validated by the manufacturer for ChIP-seq, Western blot, Immunofluorescence, and IHC in human, mouse, rat, and cow samples. According to Abcam, this polyclonal antibody "specifically detects Histone H3 acetyl-K27" and is supported by peptide-array data demonstrating strong binding to acetylated H3K27 and minimal cross-reactivity. It has also been cited in >2,000 peer-reviewed publications and successfully used in ChIP-seq assays in human leukemic cell models. Goat Anti-Rabbit IgG H&L (ab6702, Abcam) is a polyclonal secondary antibody suitable for multiple applications including Western blot, ELISA, IHC-P, IHC-Fr, IP, immunodiffusion, immunocytochemistry, and immunofluorescence. It has been cited in over 110 peer-reviewed publications, demonstrating its widespread use in research . According to Abcam, it is affinity-purified against whole rabbit IgG and specifically recognizes both heavy and light chains. This antibody has been validated by the supplier for key applications, and its cross-reactivity profile is consistent with minimal non-specific binding.

Eukaryotic cell lines

Policy information about cell lines and Sex and Gender in Research

Cell line source(s)	HEK293T (CRL-3216, ATCC), HepG2 (HB-8065, ATCC), WTC11 (GM25256, Coriell Institute).
Authentication	The cell lines were not authenticated.
Mycoplasma contamination	The cell lines were not tested for mycoplasma contamination.
Commonly misidentified lines (See ICLAC register)	No commonly misidentified cell lines were used in in the study.

Plants

Seed stocks	*Report on the source of all seed stocks or other plant material used. If applicable, state the seed stock centre and catalogue number. If plant specimens were collected from the field, describe the collection location, date and sampling procedures.*
Novel plant genotypes	*Describe the methods by which all novel plant genotypes were produced. This includes those generated by transgenic approaches, gene editing, chemical/radiation-based mutagenesis and hybridization. For transgenic lines, describe the transformation method, the number of independent lines analyzed and the generation upon which experiments were performed. For gene-edited lines, describe the editor used, the endogenous sequence targeted for editing, the targeting guide RNA sequence (if applicable) and how the editor was applied.*
Authentication	*Describe any authentication procedures for each seed stock used or novel genotype generated. Describe any experiments used to assess the effect of a mutation and, where applicable, how potential secondary effects (e.g. second site T-DNA insertions, mosiacism, off-target gene editing) were examined.*
